# Anthropogenic Landscape in Southeastern Amazonia: Contemporary Impacts of Low-Intensity Harvesting and Dispersal of Brazil Nuts by the Kayapó Indigenous People

**DOI:** 10.1371/journal.pone.0102187

**Published:** 2014-07-16

**Authors:** Maria Beatriz N. Ribeiro, Adriano Jerozolimski, Pascale de Robert, Nilson V. Salles, Biribiri Kayapó, Tania P. Pimentel, William E. Magnusson

**Affiliations:** 1 Programa de Pós-Graduacão em Ecologia, Instituto Nacional de Pesquisas da Amazônia (INPA), Manaus, Amazonas, Brasil; 2 Associação Floresta Protegida (AFP), Tucumã, Pará, Brasil; 3 Institut de Recherche pour le Développement (IRD), UMR PALOC, Paris, France; 4 Coordenação de Ciências Humanas, Museu Paraense Emílio Goeldi, Belém, Pará, Brasil; 5 Coordenação de Dinâmica Ambiental (CDAM), Instituto Nacional de Pesquisas da Amazônia, Manaus, Amazonas, Brasil; 6 Coordenação de Biodiversidade, Instituto Nacional de Pesquisas da Amazônia, Manaus, Amazonas, Brasil; New York State Museum, United States of America

## Abstract

Brazil nut, the *Bertholletia excelsa* seed, is one of the most important non-timber forest products in the Amazon Forest and the livelihoods of thousands of traditional Amazonian families depend on its commercialization. *B. excelsa* has been frequently cited as an indicator of anthropogenic forests and there is strong evidence that past human management has significantly contributed to its present distribution across the Amazon, suggesting that low levels of harvesting may play a positive role in *B. excelsa* recruitment. Here, we evaluate the effects of Brazil nut harvesting by the Kayapó Indigenous people of southeastern Amazonia on seedling recruitment in 20 *B. excelsa* groves subjected to different harvesting intensities, and investigated if management by harvesters influences patterns of *B. excelsa* distribution. The number of years of low-intensity Brazil nut harvesting by the Kayapó over the past two decades was positively related to *B. excelsa* seedling density in groves. One of the mechanisms behind the higher seedling density in harvested sites seems to be seed dispersal by harvesters along trails. The Kayapó also intentionally plant *B. excelsa* seeds and seedlings across their territories. Our results show not only that low-intensity Brazil nut harvesting by the Kayapó people does not reduce recruitment of seedlings, but that harvesting and/or associated activities conducted by traditional harvesters may benefit *B. excelsa* beyond grove borders. Our study supports the hypothesis that *B. excelsa* dispersal throughout the Amazon was, at least in part, influenced by indigenous groups, and strongly suggests that current human management contributes to the maintenance and formation of *B. excelsa* groves. We suggest that changes in Brazil nut management practices by traditional people to prevent harvesting impacts may be unnecessary and even counterproductive in many areas, and should be carefully evaluated before implementation.

## Introduction

There is a growing body of evidence supporting the hypothesis that the Amazon is a mosaic of anthropogenic landscapes, managed and domesticated by indigenous pre-Columbian people in various degrees, rather than a pristine and untouched forest [1]–[5]. *Bertholletia excelsa* (Lecythidaceae) groves, locally known as *castanhais*, are frequently cited as anthropogenic forests in the Amazon region [1], [6]. *B. excelsa* seeds, Brazil nuts, have been used for subsistence by indigenous people in the Amazon forest for thousands of years [6], and there is increasing evidence that those human populations have significantly influenced the current distribution of this species across the Amazon [6]. The occurrence of *B. excelsa* groves in many parts of the Amazon is associated with patches of Amazonian anthropogenic dark earths [1], [8] and recruitment of *B. excelsa* trees is favored in agricultural fallows and disturbed sites [9], [10]. Genetic and linguistic data also suggest that the dispersal of the species throughout the Amazon Basin is a recent process [6], [11]–[13], which cannot be explained solely by the activity of natural dispersers, and Scoles & Gribel [14] found that *B. excelsa* stands were younger in sites with more intensive pre- and post-colonial human occupation.

Brazil nuts have been harvested almost exclusively from the wild and their commercialization represents an important source of revenue for many Amazonian indigenous and riverine communities, and one of the most promising alternatives to predatory economic activities [15], [16]. Due to its uncontestable economic importance, there has been concern over the sustainability of Brazil nut harvesting [17]–[20]. While some studies have shown that medium and even high levels of harvesting may be sustainable over the long term, a meta-analysis conducted by Peres et al. [18], which was later criticized by Scoles & Gribel [20], concluded that long-term intensive Brazil nut harvesting has reduced *B. excelsa* recruitment throughout the Amazon. Despite the possible demographic impacts of high-intensity Brazil nut harvesting, the historical evidence [6], [8], [14] suggest that past human activities favored *B. excelsa* and, therefore, that low levels of harvesting may play a positive role in the recruitment of this species.

Much of the current Brazil nut harvesting in the Amazon is still carried out by traditional people, who often conduct non-intensive seed collection [19], [21]. Many of those traditional harvesters, such as indigenous peoples, have interacted with *B. excelsa* for centuries [22] and may be also intentionally and non-intentionally managing *B. excelsa* stands [23], [24]. The commercialization of Brazil nuts provides one of the major sources of income for the Kayapó Indians, a *Jê* speaking indigenous group that maintains periodic semi-nomadic practices [25] and is known to manage many natural resources [23], [26]. Kayapó informants say that ancient Kayapó villages and camping sites are associated with Brazil nut, babaçu (*Orbignya* spp.), açaí (*Euterpe* spp.) and bacaba (*Oenocarpus* spp.) plantations, and that the planting of those species is an ancient Kayapó tradition [26].

The Kayapó group includes approximately 10000 people that inhabit a Brazil nut rich territory on both sides of the Xingu River. The Kayapó territory, together with other indigenous lands and conservation reserves, forms a continuous forest corridor of 28 million ha within the arc of deforestation in southeastern Amazonia. Inhabited by 25 indigenous groups, including the Kayapó, and hundreds of riverine families, the Xingu basin is in large part considered an anthropogenic landscape, inhabited and managed by indigenous people over at least the last 1200–1500 years [27]. The Kayapó ethnic group originated in the savanna biome, in an area between the Tocantins and Araguaia rivers, located about 200 km east of the region in which it is found today [25]. The migration of the Kayapó to the west occurred at least 170 to 250 years ago [25], although they probably interacted with the Amazonian forests earlier than that. Before their arrival, the Xingu region was inhabited by several *Tupi* speaking indigenous groups [28] and there is archeological evidence of ancient occupation of the region, including ceramic remains and sites with anthropogenic dark earths [27]. Since the occupation of the Xingu basin, the Kayapó has been the main group responsible for the active protection and management of the area [25], [29], [30]. The peaceful contact with most of the Kayapó groups occurred in the 1950s [29], [30]. During the 1980s and 1990s, some Kayapó chiefs were engaged in illegal and unsustainable activities, mainly gold mining and selective logging [31]. In the last decades, however, many Kayapó villages have invested in sustainable economic activities, and the Brazil nut trade represents one of the main income-generation initiatives. Brazil nut harvesting in recent years by the Kayapó in the three villages we studied has been mostly low intensive, varying from approximately 7% to 43% of the estimated total seed stocks of the harvested groves of the villages [21].

This study investigates the contemporary effects of Brazil nut harvesting and management by the Kayapó indigenous people on the recruitment and dispersal of *B. excelsa*. Specifically, we asked if current seed harvesting by the Kayapó of three villages has affected density of seedlings in 20 *B. excelsa* groves subjected to different harvesting intensities, whether the Kayapó effectively disperse Brazil nuts along trails used to transport seeds, and if they have intentionally or unintentionally planted *B. excelsa* seeds and seedlings in their villages.

## Material and Methods

### Study site

**Figure 1:**
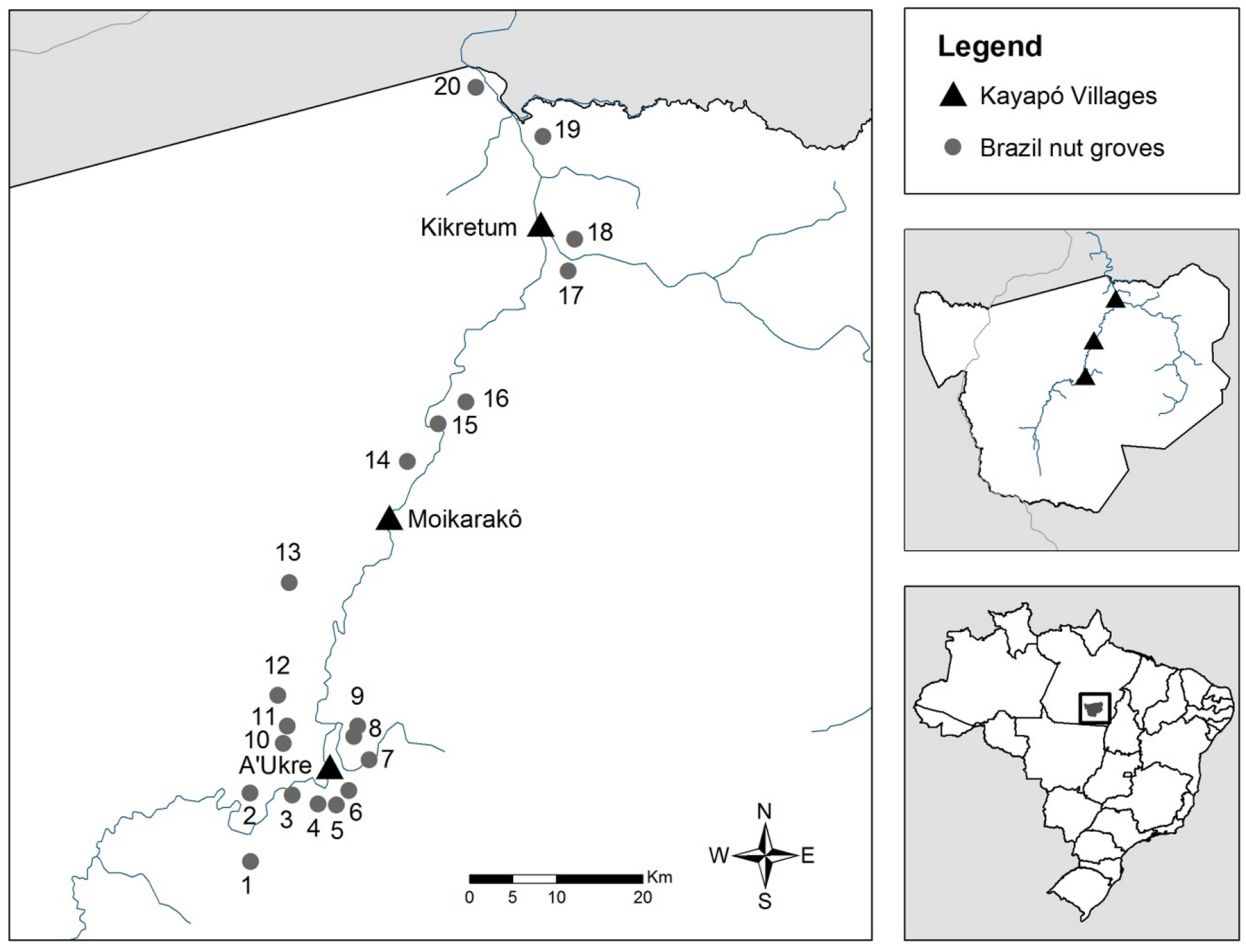
Map of the study site. The triangles indicate the location of the three Kayapó villages and the points, the location of the 20 *B. excelsa* groves sampled in this study, in Kayapó Indigenous Land, southeastern Amazonia. Identification numbers of groves correspond to those of Table S1 in the supporting information.

We conducted our study in the territories of three Kayapó villages – A'Ukre (07°41′43″S, 51°52′53″W), Moikarakô (07°26′11″S, 51°48′57″W) and Kikretum (07°08′17″S, 51°39′26″W), located along the Riozinho and Fresco Rivers, which are second- and first-order tributaries of the Xingu River, respectively. The villages are located in the Kayapó Indigenous Land, a 3.284 million ha reserve located in southern Pará State, southeastern Amazonia, in the transition between the Amazon Forest and the savannas of central Brazil (Fig. 1). The Kayapó people traditionally collect Brazil nuts for subsistence, and the close relationship between the Kayapó and *B. excelsa* is a recurrent theme in mythical, historical and present times [22]. Kikretum, A'Ukre and Moikarakô villages were founded in 1976, 1979 and 1996, respectively, and most of their families are involved in harvest of Brazil nut for subsistence and trade. A'Ukre produced Brazil nut oil for a British cosmetic company between 1991 and 2003, and has sporadically traded nuts on the local market [32]. Kikretum has constantly traded Brazil nuts on the local market, except during the gold mining and logging period between the late 1980s and 2002. Moikarakô has traded nuts on the local market only in the last few years. Since 2005, all three communities have been engaged in an initiative of Brazil nut certification and fair trading lead by the Associação Floresta Protegida (AFP), a local indigenous non-profit organization which currently represents 22 Kayapó communities.

### Study species

*Bertholletia excelsa* trees are among the tallest and most long-lived trees of the Amazon Forest. They can reach up to 50 m in height, more than 5 m in diameter at breast height [33] and may live up to 1000 years [34]. The species occurs in unflooded (*terra firme*) forests of Brazil, Bolivia, Peru, Colombia, Venezuela and the Guianas [18], [35], usually in groves of 50 to more than 300 individuals [33], [36], although individuals may be randomly distributed in the landscape at relatively low densities in some areas [37].

In the study site, *B. excelsa* groves are usually well defined, and vary greatly in area (from 5 to ≈800 ha), density of *B. excelsa* trees (1 to 5.1 individuals >60 cm DBH ha^−1^), and abundance of *B. excelsa* trees (20 to ≈800 individuals grove^−1^) [21]. Pollination depends on a few species of bees [38]. Mean *B. excelsa* annual fruit production estimated for the study site in four consecutive years varied from 103 to 269 fruits per tree (mean 184.3±72.2 fruits tree^−1^ year^−1^), but some individuals may produce up to 1000 fruits [39]. However, production varies considerably in other Amazon regions (mean 66±98 and 102 fruits tree^−1^ year^−1^ in Acre [40] and Bolivia [17], respectively). The extremely hard fruits fall during the rainy season, and contain from 8 to 30 seeds [17], [39]. Only a few species can open *B. excelsa* fruits, especially agoutis, which have been considered the main non-human *B. excelsa* seed predators and dispersers in most areas [39], [41]. Recruitment and growth of young *B. excelsa* trees is favored in open areas, such as natural forest gaps and disturbed sites [9], [42]–[44]

### Impacts of Brazil nut harvesting on seedling density

We evaluated the impacts of Brazil nut harvest on the regeneration of *B. excelsa* in 20 groves with different harvesting histories located in the territories of the Kayapó villages of A'Ukre, Moikarakô and Kikretum, including five unharvested groves. In April to June 2009 and April to June 2010, we estimated the density of *B. excelsa* seedlings and adult trees from line transects established in the groves. One transect was established in each grove from the border to the interior along the longest axis, usually crossing the entire grove. In very small groves, two perpendicular transects were established to attain a minimum length of 850 m, discounting the overlap area where they crossed. Transect lengths varied from 850 to 1400 m, depending on the size of the grove (more information on each grove is provided in the Table S1). In each line transect, we measured all individuals seen and recorded their perpendicular distance from the transect. Densities were estimated with the program DISTANCE 6.0 [45]. When the number of detected individuals in a grove was lower than 30, we corrected the density by a correction factor calculated with DISTANCE using transects of all groves pooled in a single analysis, with truncated distances of 2 m and 30 m on each side of the transect for seedlings and adults, respectively. At those truncation distances, the probability of detection of individuals calculated by DISTANCE was 100% and the mean detection distances were not different between transects (*F*_19,101_ = 1.002, *P* = 0.466 for seedlings; *F*_19,332_ = 0.051, *P* = 0.539 for adults). We considered only seedlings from 30 to 150 cm in height, since mortality rates of seedlings shorter than 30 cm are considerably higher [MBNR, unpublished data]. Adult trees were considered those with more than 60 cm diameter at breast height (DBH), since trees in the study site start to produce fruits at approximately this DBH ([39]; MBNR, unpublished data).

We defined size of *B. excelsa* groves and location of line transects with the program ArcGIS [46] from SPOT 5 HRG panchromatic satellite images with resolution of 2.5 m and SPOT 5 HRG multispectral satellite images with resolution of 10 m (details in [21]). The SPOT images allowed us to visualize harvested groves informed by the Kayapó, as well as to identify isolated and unharvested groves not known to the harvesters. One of the *B. excelsa* groves sampled was previously studied by Baider [39], who mapped all its adult individuals. Because this grove was not inside the SPOT images we had, we used the size of the grove given by Baider and defined the location of the transect from her maps. To control the effect of soil fertility on the density of *B. excelsa* seedlings, we collected a composite soil sample (1–20 cm depth) at the center of each grove. Soil analyses were conducted at the Laboratory of Soils and Plants of the Instituto Nacional de Pesquisas da Amazônia (INPA), according to standard procedures [47]. The sum of the extractable bases Ca, Mg and K (cmolc kg^−1^) was used as a measure of soil fertility.

We estimated Brazil nut harvest intensity in each *B. excelsa* grove from information on recent commercial production of nuts by the Kayapó; Smith's S index of salience, which reflects the importance of each *B. excelsa* grove for villagers; and from the number of years of commercial Brazil nut harvest in each grove during the last 20 years. Twenty years is the approximate time in which seedlings should attain a height of 150 cm based on a study of growth of 139 seedlings over two years in 12 *B. excelsa* groves in Kayapó Indigenous Land (mean growth rate of seedlings including regrowth after damage caused by falling branches was 6.3 cm year^−1^±10.5 (SD) [MBNR, unpublished data]. Recent commercial Brazil nut production in tons by the Kayapó was obtained from AFP records of 2008/09 and 2009/10 harvests, which were based on the number of sacks collected by each Kayapó harvester in each grove. Smith's S is an index of psychological salience which weights both rank and frequency of citations of an item in free listings [48], [49]. During structured interviews with 37, 32 and 29 Kayapó from A'Ukre, Moikarakô and Kikretum, we conducted free listings of the *B. excelsa* groves in the territories of each village. During interviews, the Kayapó were asked to cite the groves of their respective village. All interviews were conducted between October 2007 and February 2009 in the Kayapó language, with the help of a Kayapó translator. Smith's S index had a high correlation with commercial Brazil nut production (*r* = 0.82, *P*<0.001), and was excluded from the analysis to avoid colinearity. However, the high correlation indicates that the short-term data on commercial seed production reflects long-term harvesting. We estimated the number of years of commercial harvest during the last two decades from historical harvesting information obtained from structured interviews with Kayapó collectors, Kayapó organizations, Fundação Nacional do Índio (FUNAI) and Kayapó-elder informants. Summarized data for each *B. excelsa* grove is available in Table S1.

We used multiple regression to test the effect of Brazil nut commercial production, number of years of commercial harvest of the grove, area of the grove, soil fertility (sum of bases) and density of adult trees on the density of seedlings. Our evaluation was conducted in a single geographic area, with similar climatic conditions, so there was no variation in rainfall and temperature that might confound our conclusions about the effect of other variables.

### Dispersal of *B. excelsa* by the Kayapó

To determine the amount and frequency of *B. excelsa* seed dispersal by the Kayapó along trails, we followed 60 harvesters from 36 different families in Brazil-nut-collection trips in the three Kayapó villages, over 19 days, in 2009 and 2010, and visually counted all seeds dropped by them between the groves and camp sites. To test if Kayapó seed dispersal enhances the density of *B. excelsa* seedlings along the trails, we compared the density of seedlings from 30 to 150 cm in height in one transect along each of the three main trails used for transporting Brazil nuts from the closest *B. excelsa* groves to A'Ukre village, with the density of seedlings in a similar transect parallel to, and 100 m from each trail. We counted seedlings within 2 m on both sides of each transect. Transects were established only in old growth forests, outside *B. excelsa* groves, and began 200 m from the edge of the grove. Transect length varied from 700 to 1000 m, which was the maximum distance between groves and agricultural sites around the villages or the river. We used paired t-tests to compare density of seedlings in transects near and far from trails.

We tested if seedling growth and mortality rates were different beside trails (≤2 m) and away from trails (>2 m) from a two year demographic study with 138 seedlings from 30 to 150 cm in height (70 near trails and 68 away from trails) inside eight *B. excelsa* groves in the three villages, four of them located in the territory of A'Ukre village, one in the territory of Moikarakô village and three in the territory of Kikretum village. In March and April 2008, we measured and marked seedlings found within groves with aluminum tags and registered their geographical coordinates with a GPS. All individuals were surveyed between April and June 2010 and re-measured when alive. We used paired t-tests to compare growth and mortality of seedlings near and far from trails inside each grove.

To investigate if the Kayapó plant *B. excelsa* seeds and seedlings, we conducted interviews and surveyed for *B. excelsa* individuals in the villages. During structured interviews, we asked Kayapó villagers if they had planted or knew about ancestors who had planted *B. excelsa* trees. To determine whether *B. excelsa* individuals are planted in the villages, we conducted one-day surveys for *B. excelsa* trees in the central area and home gardens of the three Kayapó villages, which are distant at least 2.0 km from the nearest *B. excelsa* grove. Agricultural sites and secondary forests near the villages were not surveyed.

### Ethics Statement

Approval for interviewing the Kayapó was obtained from the Instituto Nacional de Pesquisas da Amazônia's Committee for Research Ethics (protocol n° 0154/07). Written informed consents were obtained from the Kayapó after meetings in each village to clearly explain in the Kayapó native language the objectives and importance of this study, as well as to clarify the content of the informed consent in order to guarantee that all villagers fully understood their right to choose to participate or not in the interviews and the confidentiality of their identity. Authorization to access the Indigenous Reserve during field work was obtained from National Indian Foundation (FUNAI, protocol n° 0313/07), the Brazilian government agency responsible for the establishment and implementation of most policies related to indigenous peoples. No traditional knowledge was accessed in this research.

## Results

### Impacts of Harvesting on Brazil Nut Seedling Density

**Figure 2:**
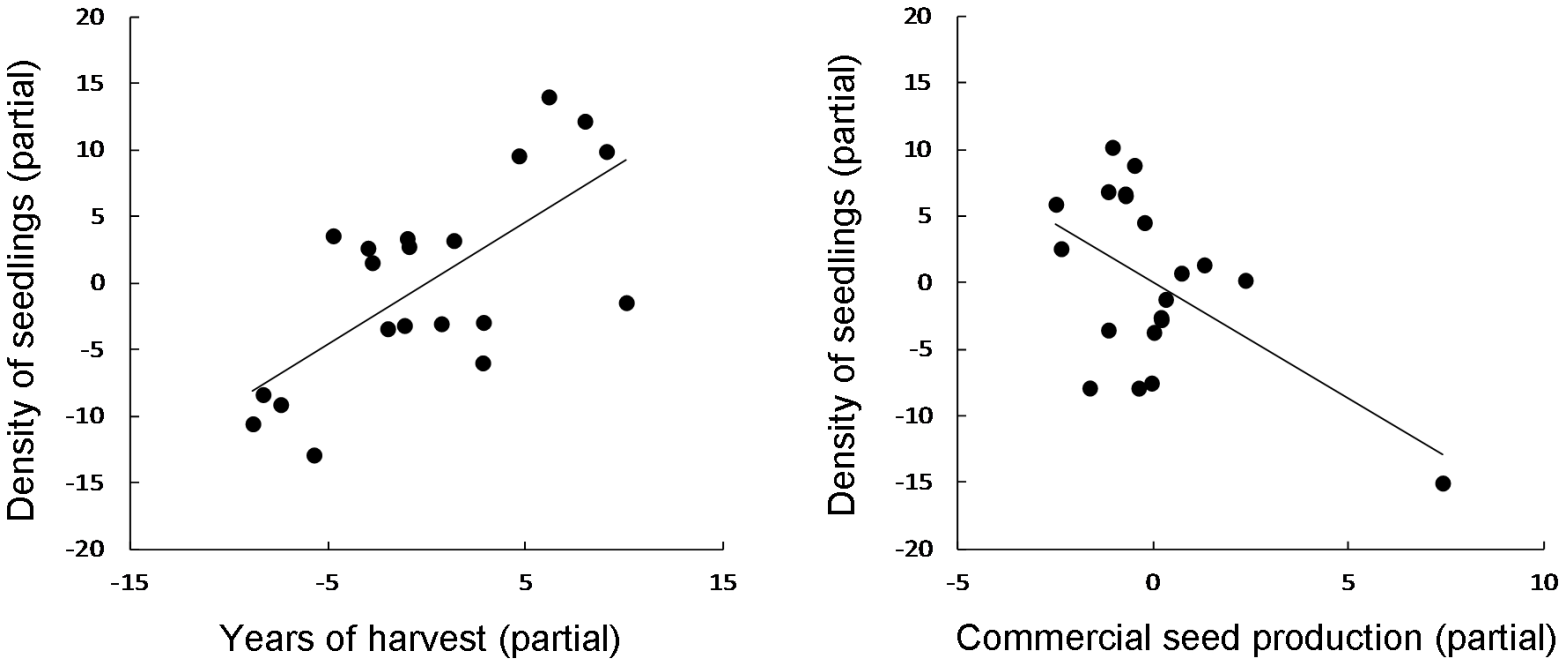
Multiple regression analysis testing the effect of Brazil nut harvesting intensity on the density of seedlings. The partial regression to the left shows the relationships between density of *B. excelsa* seedlings (DS) and years of harvest (YH), and the partial regression on the right shows the relationship between density of seedlings and commercial seed production in tons (PR) in *B. excelsa* groves in the Kayapó Indigenous Land, southeastern Amazonia. The points in each graph represent the 20 *B. excelsa* groves sampled. The full multiple regression model has *R^2^* = 0.576.

There was a strong positive effect of the number of years of Brazil nut collecting on recruitment of seedlings, despite seed losses due to harvesting (*R^2^* = 0.576, *F*_5,14_ = 3.81, *P* = 0.022). The multiple-regression model relating density of seedlings (DS) to Brazil nut commercial production in tons (PR), years of harvest of the grove (YH), area of the *B. excelsa* grove (AG), soil fertility (SF) and density of adult trees (DA) (DS = 5.648 - 1.747PR+0.922YH+0.015AG - 0.152FS+0.793DA) accounted for 58% of the variance in the density of seedlings (Table 1). There was little evidence of colinearity (tolerance >0.51 for all variables). Only years of harvest (*P* = 0.003) and commercial production (*P* = 0.027) contributed significantly to the model (Fig. 2). However, models containing only years of harvest and commercial production indicated that the effect of years of harvest was much stronger than that of production. A model with only years of harvest (*r^2^* = 0.36) explained 15% less variance than the model with both variables (*R^2^* = 0.51). A model with only production had an *r^2^* of only 0.04. If we remove the *B. excelsa* grove in which the most seeds have been collected (Piykôny, number 4 in Fig. 1 and Table S1), the negative effect of commercial production on density of seedlings is no longer significant.

**Table 1:** Results of multiple regressions testing the effects of Brazil nut harvesting on the density of *B. excelsa* seedlings in 20 *B. excelsa* groves located in Kayapó Indigenous Land, southeastern Amazonia.

Effect	Coefficient	Std. Error	Std. Coef.	Tolerance	*t*	*P*
Constant	5.648	4.797	0.000	-	1.177	0.259
Commercial production	−1.747	0.708	−0.597	0.518	−2.468	0.027
Years of harvest	0.922	0.261	0.696	0.781	3.537	0.003
Area of the grove	0.015	0.011	0.336	0.508	1.376	0.190
Soil fertility	−0.152	0.421	−0.064	0.964	−0.361	0.723
Density of adult *B. excelsa* trees	0.793	1.346	0.121	0.719	0.589	0.565

### Management of *B. excelsa* by the Kayapó

**Figure 3:**
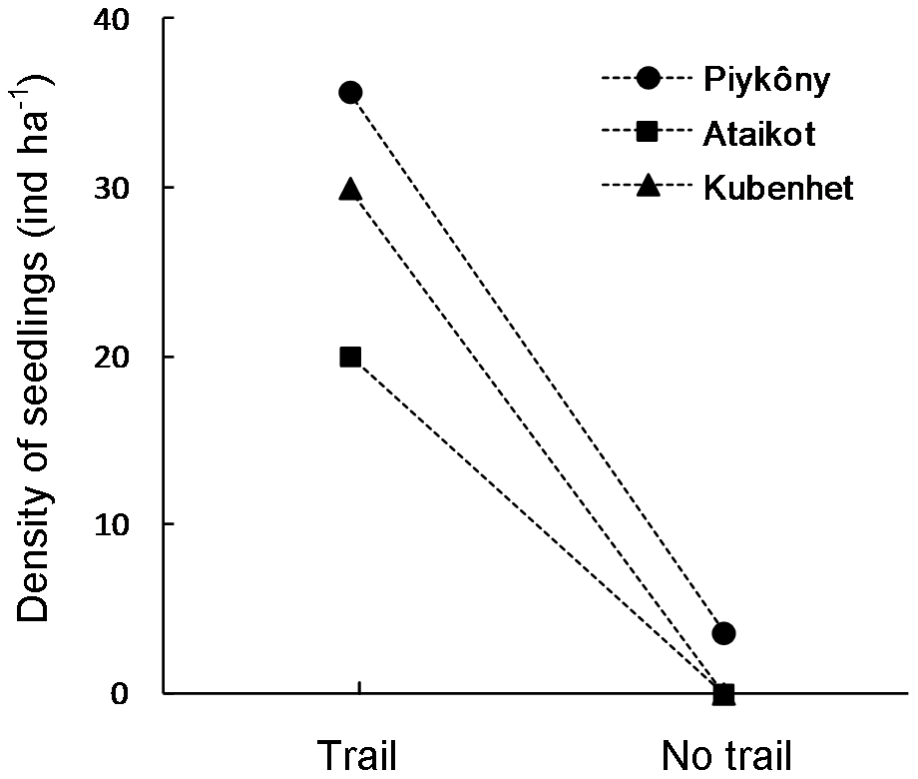
Comparison of *B. excelsa* seedling density along trails and in parallel transects away from trails. Each point represents one of the transects in the territory of A'Ukre village, Kayapó Indigenous Land, southeastern Amazonia. The three points on the left are transects located along the main trails used to transport Brazil nuts from groves to the village and the three points on the right are transects parallel to those trails and 100m from them. Points with the same symbol represent pairs of along-trail and away-from-trail transects.

Kayapó collectors were seen to drop a total of 34 *B. excelsa* seeds (1.8 day^−1^) along trails on 16% of the days they were followed. Moreover, density of *B. excelsa* seedlings (Fig. 3) within 2 m of the trails (mean  = 28.6 seedlings ha^−1^ ±7.09 SD) was much higher (paired t-test: *t*_2_ = 7.33, *P* = 0.018) than along the parallel transects 100 m from the trails (mean  = 1.2 seedlings ha^−1^, ±2.1 SD). Estimated density of seedlings along trails outside groves was twice the density of seedlings of all *B. excelsa* groves sampled in this study (13.4 ha^−1^ ±8.5 SD) and more than four times the density of seedlings in the five unharvested groves (6.4 ha^−1^±4.5 SD).

Mean growth rate, including reduction in size due to cutting or other damage, and mortality of seedlings within 2 m of trails in groves (mean  = 9.7 cm±12.7 SD and 3.3%±5.1 SD, respectively) were not significantly different (paired t-test: *t*_7_ = 0.447, *P* = 0.669 for mean growth rate; *t*_7_ = 0.586, *P* = 0.576 for mortality) from those of seedlings located away from trails (11.2 cm±9.2 SD for mean growth rate; 4.6%±5.4 SD for mortality).

Of the 98 villagers interviewed, 20.4% declared that they had intentionally planted at least one *B. excelsa* seedling in the village or agricultural fields and 41.7% said that their ancestors or older relatives planted *B. excelsa* trees. We located six, eight and 51 *B. excelsa* individuals within Moikarakô, Kikretum and A'Ukre villages, respectively. Most of them were seedlings between 30 and 150 cm in height (41.5%), or saplings with DBH <10 cm (30.8%). Three individuals were already producing seeds. According to information obtained from the Kayapó, most individuals found were intentionally planted by the villagers, while others germinated from seeds unintentionally dropped in their home gardens.

## Discussion

Although we cannot predict the effect of a future possible intensive seed collection on *B. excelsa* recruitment in the study site, the current low-intensity harvesting conducted by the Kayapó did not reduce seedling recruitment in *B. excelsa* groves. Despite the large quantities of Brazil nuts collected, the Kayapó have only harvested a small proportion (∼7% to 43%) of the total seed production of harvested groves in the recent years [21], which is lower than the levels of harvesting described as sustainable in other studies [17], [19]. In fact, it has been suggested that even high levels of fruit or seed harvesting of some trees may allow persistence of the species in the area over the long term [50].

Our study also showed that the number of years of harvesting conducted by the Kayapó and/or other activities associated with it increased the density of seedlings in *B. excelsa* groves. This increase is probably a result of several non-exclusive mechanisms. Demographic mechanisms, such as density-dependent mortality [51], [52], could be responsible for the increase in seedling density due to a decrease in seed density and, consequently, a reduction in seed and seedling attractiveness to predators or pathogens [53], [54]. Furthermore, besides Brazil nut collection, the most accessible *B. excelsa* groves are also used by harvesters to hunt and collect other forest products, such as fruits, honey, medicines and fibers. Even considering that Brazil nut harvesting and those extractive activities have a low impact in forest structure, they may result in small disturbances (e.g. trails) which could increase light levels in the forest understory, favoring regeneration of *B. excelsa* (42–44). It is also possible that hunting conducted by the Kayapó in the harvested sites could enhance seedling recruitment by reducing density of *B. excelsa* seed and/or seedling predators, or influence patterns of *B. excelsa* seed dispersal in these sites [55]–[57]. Density of game species in the vicinity of one of the studied Kayapó villages (A'Ukre), which includes frequently harvested *B. excelsa* groves, was previously shown to be lower than in remote areas [58].

Despite the possible importance of other mechanisms, the extremely high density of seedlings along trails used to transport Brazil nuts to the village indicates that the efficiency of Kayapó harvesters as *B. excelsa* seed dispersers is one of the reasons for the high density of seedlings within most harvested groves. *B. excelsa* groves have a complex and dynamic net of trails between harvested trees and, considering that the harvesting season lasts at least two months per year, and that several Kayapó families collect and transport Brazil nuts simultaneously, the annual number of seeds dispersed along trails must be substantial. Kayapó collectors carry *B. excelsa* seeds long distances (kms) within and outside groves. Although scatter-hoarding rodents, which can move seeds for considerable distances (up to 50 m) from the mother trees [41], [59], have been cited as the main agents responsible for the current spatial distribution of *B. excels*a [36], human harvesters can disperse seeds over distances much greater than can be expected for these small rodents with home ranges of a few hectares [60], [61]. Therefore, seeds dispersed by harvesters could benefit from lower predation due to the higher distance from the mother tree [51], [52], [62], and from greater chance of germination if they are dispersed to suitable sites [53], [63]. Although seedlings along trails may be more prone to be cut by harvesters, growth and mortality rates were similar near trails and far from trails. Seedlings from seeds dispersed along trails may grow faster due to higher light levels [42], which possibly compensates for the negative effects of being cut.

The unintentional dispersal of seeds by the Kayapó along trails between groves, camps and villages indicates that seed dispersal along trails is a potential mechanism to expand or initiate new groves. Moreover, *B. excelsa* is intentionally and unintentionally planted by the Kayapó in villages and agricultural sites, and we found several *B. excelsa* individuals in the villages. Considering that those villages are less than 35 years old, that the Kayapó have inhabited the Xingu basin region and harvested Brazil nuts for at least 170 years, and that many Kayapó villages have changed their locations several times [25], it is likely that *B. excelsa* planting by the Kayapó has significantly influenced the *B. excelsa* spatial distribution within their territory. This finding corroborates information obtained by Posey [26] that ancient Kayapó villages are associated with Brazil nut groves and that the Kayapó traditionally plant Brazil nut trees. Brazil nut harvesting by the Kayapó may represent an unusual case of harvesting associated with management, but since there is apparently no specific or complex traditional knowledge used by the Kayapó to manage *B. excelsa*, similar responses to Brazil nut harvesting by indigenous and non-indigenous people is probably also occurring in several other Amazonian regions [20], [24], [35].

Together, our results support the hypothesis that *B. excelsa* dispersal throughout the Amazon was, at least in part, influenced by pre-Colombian indigenous groups [1], [6], corroborating the theory that the Amazon forest is a mosaic of landscapes modified by humans in the past [2], [4]. Moreover, current human management probably still contributes to the maintenance and formation of *B. excelsa* groves in the Xingu basin, and possibly in other Amazonian sites. This may have significant implications for the management of *B. excelsa* and raises an important question: If *B. excelsa* groves are indeed anthropogenic formations, is some level of human interference needed for their maintenance in the long term or is the activity of scatter-hoarding rodents by itself [36], [41] enough to maintain the dynamics of groves at a landscape scale? This might have implications for the long-term conservation of *B. excelsa*, especially inside Amazonian reserves that do not allow the presence of human settlements.

There has been much discussion about how to conciliate the needs of local people and conservation priorities in the Amazon region [31], [64], [65], and one of the priority actions to advance in this question and move towards a more inclusive conservation policy is to assess the ecological impacts of the use of natural resources by traditional populations [66]. Here we have shown that harvesting of Brazil nuts by the Kayapó people of southeastern Amazonia, as practiced today, is an example of beneficial coexistence between people and a natural resource. We still do not know the effects of a possible future increase in Brazil nut market demand, and consequently in harvesting intensity, on the recruitment of *B. excelsa* inside groves of the Kayapó territories. However, so far, Brazil nut harvesting not only has provided livelihoods for the Kayapó, contributed to the maintenance of their culture and reduced the attractiveness of predatory sources of income, such as logging and mining, but it has also positively affected *B. excelsa* recruitment and dispersal. We recommend that any suggestions for changes to *B. excelsa* management practices by traditional people aimed to reduce harvesting impacts (e.g. [18]), should be carefully evaluated and tailored to different social and environmental contexts. Management prescriptions based on broad generalizations may disrupt the delicate equilibrium between use and conservation of this valuable resource by local Amazonian communities.

## Supporting Information

Table S1 **Information on** ***Bertholletia excelsa*** **groves sampled in Kayapó Indigenous Land, southeastern Amazonia.**
(DOCX)
