# Supplementary material for: Anthropogenic Landscape in Southeastern Amazonia: Contemporary Impacts of Low-Intensity Harvesting and Dispersal of Brazil Nuts by the Kayapó Indigenous People
Source: PLoS One. 2014 Jul 16;9(7):e102187. doi: 10.1371/journal.pone.0102187 (PMC4100818; doi:10.1371/journal.pone.0102187)
Supplement: Table S1 — Information on Bertholletia excelsa groves sampled in Kayapó Indigenous Land, southeastern Amazonia. (DOCX) [file pone.0102187.s001.docx]

**Supporting information**

**Table S1.** **Information on *Bertholletia excelsa* groves sampled in Kayapó Indigenous Land, southeastern Amazonia.**

| *Bertholletia excelsa* grove | Kayapó village | Density of  *B. excelsa* seedlings 30-150cm high (ind ha^-1^) | Recent commercial production (tons) | Years of harvest in the last 20 years | Area of the grove (ha) | Soil fertility -sum of bases (cmolc kg^-1^) | Density of  *B. excelsa* trees of DBH>60cm (ind ha^-1^) | Length of transect (m) | Smith’s S Salience Index |
| --- | --- | --- | --- | --- | --- | --- | --- | --- | --- |
| 1. Pinkaiti | A'Ukre | 7.9 | 0.00 | 0 | 28.5 | 2.01 | 3.7 | 950 | 0.0 |
| 2. Pinkaiti ny | A'Ukre | 12.5 | 0.00 | 0 | 82.0 | 3.04 | 1.0 | 1000 | 0.0 |
| 3. Kikrérytí | A'Ukre | 28.9 | 0.30 | 14 | 25.8 | 2.88 | 3.4 | 950 | 0.1 |
| 4. Piykôny * | A'Ukre | 5.4 | 10.81 | 16 | 236.6 | 1.73 | 3.6 | 1400 | 0.5 |
| 5. Bemp | A'Ukre | 17.5 | 0.00 | 14 | 47.8 | 0.92 | 5.1 | 1000 | 0.1 |
| 6. Atâikot Piykô * | A'Ukre | 25.0 | 1.10 | 16 | 232.0 | 2.70 | 2.6 | 900 | 0.2 |
| 7. Kubenhet nho Piykô * | A'Ukre | 10.5 | 0.26 | 16 | 25.8 | 8.88 | 3.0 | 950 | 0.2 |
| 8. A'Ukre Velho | A'Ukre | 28.9 | 0.60 | 16 | 37.8 | 0.56 | 4.9 | 950 | 0.2 |
| 9. A'Ukre II | A'Ukre | 26.3 | 0.68 | 16 | 39.9 | 8.59 | 3.9 | 950 | 0.2 |
| 10. Piôkrótíkôyagot | A'Ukre | 5.9 | 0.00 | 0 | 30.0 | 9.21 | 4.7 | 850 | 0.0 |
| 11. Picadinha | A'Ukre | 5.6 | 0.00 | 0 | 23.5 | 5.75 | 3.6 | 900 | 0.0 |
| 12. Kroat | A'Ukre | 0.0 | 0.00 | 0 | 38.6 | 2.66 | 2.4 | 1050 | 0.0 |
| 13. Moikarakô Txêt | Moikarakô | 17.3 | 0.00 | 1 | 80.0 | 0.40 | 2.2 | 1300 | 0.0 |
| 14. Nhandjúbindjã | Moikarakô | 16.0 | 0.23 | 5 | 25.0 | 9.81 | 4.1 | 1250 | 0.1 |
| 15. Oredjã | Moikarakô | 6.8 | 1.89 | 6 | 141.5 | 3.18 | 1.4 | 1100 | 0.3 |
| 16. Pintekrébikiere | Moikarakô | 10.0 | 5.34 | 6 | 346.6 | 12.16 | 1.7 | 1000 | 0.6 |
| 17. Urubu | Kikretum | 12.5 | 5.06 | 8 | 215.0 | 5.31 | 2.2 | 1000 | 0.4 |
| 18. Kentí | Kikretum | 15.0 | 5.89 | 8 | 840.0 | 1.80 | 2.2 | 1000 | 0.5 |
| 19. Capoeira | Kikretum | 4.2 | 1.74 | 8 | 146.0 | 2.01 | 1.3 | 1200 | 0.1 |
| 20. Rebojinho | Kikretum | 12.5 | 4.10 | 8 | 282.0 | 1.35 | 1.0 | 1000 | 0.1 |

* *B. excelsa* groves whose trails to the village (and parallel transects) were searched for seedlings.
